# Supplementary material for: Are carbon emissions trading and green financial instruments synergistic? -Comprehensive quantitative research based on content analysis
Source: PLoS One. 2024 Mar 7;19(3):e0298601. doi: 10.1371/journal.pone.0298601 (PMC10919601; doi:10.1371/journal.pone.0298601)
Supplement: S1 Data — (ZIP) [file pone.0298601.s001.zip › Data/地级市Z_F工作报告.zip!/║┌┴·╜¡/╝a╬≈╩╨/└┤╫╘╣1⁄2╓┌║┼í╛┬φ┐╦╩2╛▌═°í┐_2016.docx]

各位代表：

现在，我代表市政府向大会作工作报告，请予审议。

一、2015年工作回顾和“十二五”主要成就

2015年，面对经济下行的巨大压力，在市委的坚强领导下，在市人大、市政协的监督支持下，全市上下团结一心、迎难而上、砥砺奋进，较好地完成了年初确定的目标任务。

——经济运行保持平稳。预计，地区生产总值增长3.5%，规模以上工业增加值增长2.6%，固定资产投资增长7%，社会消费品零售总额增长8.5%，一般公共预算收入增长2.4%，城乡居民人均可支配收入分别增长5%和10%，以上指标都完成了年初预期目标。外贸进出口总额下降22%。

——项目建设推进有力。全年实施投资5000万元以上重点产业项目104项，当年完成投资44亿元。北汽普莱德锂电负极材料、青岛啤酒搬迁扩建、汇源集团新生态乳业基地等一批新开工和续建项目加快建设。新科120万吨洁净煤、月亮岛水上乐园一期等一批项目建成投产，为鸡西转型发展注入了动力，增添了后劲。

——“三大战略”深入实施。农业“两个转变”再创佳绩。粮食喜获丰收，总产量61.9亿斤。绿色食品种植基地监测面积500万亩，绿色、有机、无公害农产品标识216个，农业产业化龙头企业达到88户。畜牧业实现产值49亿元，增长9%，新建汇源、娃哈哈千头牛场2处、万头猪场3处，国家级标准化示范场2处。工业“双轮驱动”提档增速。“两黑一绿一药”四大产业加速成长，规上企业实现产值155亿元，占工业比重74.5%。实施企业裂变升级项目45个，完达山酒业、奥星石墨等一批企业实现扩张发展。珍宝岛药业成功上市，开创了本土企业A股上市融资先河。国家级石墨检测检验中心顺利通过验收，举办了2015中国石墨产业发展论坛，“中国石墨之都”的影响力大幅提升。三产“旅贸牵动”成效显著。兴凯湖、乌苏里江、珍宝岛湿地列入省骨干景区，全年旅游业总收入增长32.6%。口岸进出口货物和出入境人员分别增长66.2%、15.7%，中俄(鸡西)义乌小商品展、青岛啤酒节等活动，有效拉动了居民消费。金融机构贷款余额583.2亿元，有力支持了地方经济建设。

——对上争取再创新高。全年争取资金84亿元，增长21.7%。经过不懈努力，我市被纳入国家资源枯竭型城市转移支付补助范围；鸡西历史上投入最多、改造规模最大的民生工程——采煤沉陷区棚户区改造工程，得到国家大力支持；鸡冠老工业区和恒山、滴道独立工矿区搬迁改造一期国家资金到位，一批对上争取项目为转型发展提供了动力。全年引进国内到位资金303.6亿元，同比增长10%；签约项目184个，同比增长48.4%。实际利用外资1.5亿美元，同比增长10%。

——城市功能不断完善。编制完成《鸡西城市色彩规划》，确定了“碧水蓝天映辉煌”的城市色彩概念主题。开工建设了穆棱河众兴大桥和左家桥二期，完成了文化路拓宽改造和鸡图路平岗至梨树段大修。改造中心区老旧供热管网70.8公里，恢复供气区域21个，覆盖居民2.38万户。开工建设了垃圾焚烧发电和宝川垃圾处理厂改扩建工程，三县(市)六区9座污水处理厂全部投入使用。

——民生保障富有成效。全年用于民生支出102亿元，占一般公共预算支出的69%。15件民生实事件件落实，其中，新开工采煤沉陷区棚户区改造项目8个、12979套，货币化安置1万户，城市棚户区改造项目东升花园多层竣工入住，森林名苑高层主体完工；改造弃管楼289栋、背街巷道117条，城市森林公园建成开放。多方筹措资金，15.8万名企业退休人员养老金标准月人均提高220元；城乡居民医保年人均补助标准提高到380元；市城乡低保标准分别提高到月人均500元和年人均3500元；市直单位住房公积金缴存比例提高到12%，达到国家上限；调整机关事业单位工作人员工资标准，月人均增资430元。建成农村分布式能源3163户。支持龙煤集团稳岗就业，分流安置富余人员5851人。

——社会事业全面进步。新建校舍1.45万平方米，完成教育“三通两平台”建设，资助贫困学生3.5万人次。市中医院新址投入使用，建成标准化社区12个、中心村文化活动室和文化广场71个。成功举办龙江首届“多威杯”公路拉力赛等体育赛事。完成了阶梯水价改革、供热价格调整。安全生产和社会治安持续平稳。

——发展环境持续优化。扎实开展“三严三实”专题教育，推动了政府机关作风转变。完成“两个清单”制定和“四证合一”、“一照一码”登记制度改革，清除126项非行政许可审批事项，市级行政审批事项精简到147项，是目前全省行政审批事项最少的城市。梳理优化市级行政权力流程，编制完成一次性告知单和权力运行流程图，行政权力审批要件减少89个，行政许可办理时限压缩20%，网上集中审批事项办结率97%。

各位代表，“十二五”时期是我市发展进程中不平凡的五年。这五年，综合实力不断增强。2015年，全市地区生产总值预计实现513亿元，是2010年的1.2倍。人均地区生产总值由2010年的22083元提高到28033元。固定资产投资五年累计完成1180亿元。这五年，转型发展稳步推进。三次产业结构由“十一五”末的25.6：42.3：32.1调整为37：25：38。共实施市级重点产业项目475项，累计完成投资436亿元。先后引进北汽、汇源等一批行业领军企业，为发展增添了动力。这五年，城市面貌大幅改观。实施了城市供水、供热、供气等工程。新建穆棱河大桥、团结立交桥、左家立交桥和滴道公铁立交桥，鸡虎高速、鸡兴路、南外环路竣工通车。建成了穆棱河公园和东山公园。这五年，生态建设成效显著。兴凯湖流域生态环境治理上升为国家战略，穆棱河被国家确定为生态流域补偿试点，造林绿化、退耕还林49.2万亩，治理水土流失130万亩，全面完成“十二五”节能减排任务。这五年，人民福祉持续提升。全市用于民生支出累计371亿元，年均增长9.4%。完成各类棚户区改造553万平方米，建设廉租房、公租房、经济适用房23万平方米，改造农村泥草房239万平方米。城乡居民人均可支配收入年均分别增长9.4%、15.1%。

各位代表，这些成绩的取得，是市委统揽全局、正确领导的结果，是市人大、市政协及社会各界有效监督、鼎力支持的结果，是全市上下戮力同心、拼搏奋进的结果。在此，我代表市政府向全市广大干部群众，向所有关心、支持鸡西发展的同志们、朋友们，表示衷心的感谢，并致以崇高的敬意！

在看到成绩的同时，我们也清醒地认识到存在的困难和问题。经济总量小，下行压力大；资源型城市结构性矛盾依然突出，产业转型升级步伐不快；煤炭市场持续低迷，涉煤企业生产经营困难；立市、立县(市)区、财税贡献率高的大项目少；保障和改善民生任务繁重；发展环境与人民群众的期望和发展要求有不小差距，等等。这些问题，我们一定高度重视，倾力解决。

二、“十三五”时期奋斗目标及2016年工作任务

“十三五”时期，是我市推进转型发展、实现兴市富民目标的攻坚时期，是实现全面建成小康社会奋斗目标的决胜时期。

“十三五”时期，鸡西经济社会发展的指导思想是：全面贯彻党的十八大以来中央和省委各项决策部署，深入贯彻习近平总书记系列重要讲话精神，按照中央“五位一体”总体布局和“四个全面”战略布局，牢固树立“创新、协调、绿色、开放、共享”发展理念，深入落实省委“五大规划”和“龙江丝路带”规划，围绕实现全面建成小康社会目标，突出“一条主线”，实施“三大战略”，做强“四大主导产业”，统筹推进经济、政治、文化、社会、生态文明和党的建设，不断开创“转型发展兴市富民”新局面，努力创造鸡西人民更加幸福美好生活。

发展目标：到2020年，地区生产总值和城乡居民人均收入在2010年基础上翻一番，“十三五”期间年均分别增长8%和7%；规模以上工业增加值年均增长15%—20%；一般公共预算收入年均增长5%；固定资产投资年均增长10%；社会消费品零售总额年均增长8%；外贸进出口总额年均增长5%。

2016年是“十三五”开局之年，市政府工作的总体要求是：全面贯彻落实市委十二届七次全会精神，以“转型发展兴市富民”为目标，按照“五个不动摇、四个协调发展”的要求，深入实施“三大战略”，做强“四大主导产业”，着力建设“一都五基地”，坚持上项目增实力，强产业稳增长，惠民生促和谐，重安全保稳定，更加注重结构调整，更加注重改革开放，更加注重创新创业，更加注重环境优化，推动经济社会持续健康发展。

经济社会发展的主要预期目标：地区生产总值增长6.5%—7%，规模以上工业增加值增长5%，固定资产投资增长7%，一般公共预算收入增长3%，社会消费品零售总额增长8%，外贸进出口总额增长5%，城乡居民人均可支配收入分别增长5%、8%。节能减排指标达到省核定标准。

为实现上述目标，重点做好以下八个方面工作：

(一)全力推进项目建设，在集聚发展动能上实现新提升

加快项目建设进度。全年启动实施投资5000万元以上产业项目110个，当年计划完成投资102亿元。围绕资源精深加工，引进一批项目，重点抓好投资80亿元的韩国GS集团石墨显示面板、10亿元的裕同集团印刷包装、8.4亿元的深圳红源集团中俄商贸交流中心、4亿元的安徽楚江集团石墨深加工、上海久江集团石墨深加工等项目的对接与落地。围绕扩大投资，开工建设一批项目，重点抓好投资10亿元的滴道太阳能光伏发电、7亿元的翰沃集团家居建材高端商场、3亿元的福建绿宝集团现代农业物流园等项目，形成新的经济增量。围绕产业链延伸，裂变生成一批项目，重点抓好投资20亿元的北汽集团锂电池芯系统集成、10.5亿元的贝特瑞负极材料扩能和活性炭、5亿元的珍宝岛药业三期等项目建设，提高投资强度。围绕做大经济总量，竣工投产一批项目，重点抓好投资6.3亿元的青岛啤酒搬迁扩建一期、5.7亿元的福娃稻米产业园、3.5亿元的汇源新生态乳业等38个项目。

加大招商引资力度。以“两黑一绿一药”产业为重点，紧盯国内500强和行业领军企业，突出专题招商、定向招商、以商招商，加大55个投资10亿元以上谋划项目的招商力度，力争落实一批投资主体。更加注重招商的质量和效益，积极引进一批科技含量高、财税贡献大、带动能力强、吸纳就业多的优质项目。落实《鸡西市关于支持重点产业重点项目发展的实施意见》，用好产业和项目发展引导资金，提升招商引资吸引力。抓住国家实施新一轮振兴东北等老工业基地战略契机，继续加大对上争取力度，努力争取到上级更多的政策、项目和资金支持。

加强重点园区建设。坚持项目在园区落地、要素向园区集聚，推动产业在园区集群发展。进一步完善鸡西石墨产业园区基础设施建设，明确恒山、麻山园区产业分工，推进园区产业配套发展。积极推动煤化工产业园区建设，完成A区1.24平方公里净地和B区四、六区土地征收。鸡冠产业园区收储土地500亩。调整创新园区管理体制机制，提高管理服务水平。

(二)全力推进“双轮驱动”，在做大主导产业上实现新提升

推动四大产业扩张上增量。围绕“两黑一绿一药”四大产业，做好延链、扩链文章，推进产业向中高端迈进。石墨深加工产业，以打造“中国石墨之都”为引领，依托普莱德、贝特瑞等企业，加快建设锂电负极材料项目，打造蓄能材料产业链；依托浩市、瑞诺恒等企业，加快建设人造金刚石项目，打造超硬材料产业链；依托金宇、申太等企业，加快建设膨胀石墨、石墨纸项目，打造密封材料产业链；紧盯石墨烯技术研发前沿，引进建设石墨烯加工项目，推进石墨烯制品产业链发展。同时，推进人才、标准、交易、检测、研发等基地建设，助推石墨产业向高端发展。煤化工产业，以打造现代煤化工基地为引领，围绕煤制烯烃、芳烃、乙二醇和煤制天然气等产业链谋划项目，加强与中石化集团、神雾集团的沟通衔接，做实项目前期，促成项目落地。绿色食品加工产业，以打造绿色食品生产加工基地为引领，着力发展绿色有机农产品、高品质乳制品、优质肉制品、特色山产品四大产业链，重点推进汇源新生态乳业、娃哈哈奶制品深加工、中玉食品玉米精深加工等项目，进一步壮大绿色食品产业群体。生物医药产业，以打造生物医药制造基地为引领，支持引导珍宝岛、乌苏里江、新医圣等企业裂变发展，做大做强；大力推广刺五加、桔梗、丹参等北药种植，谋划建设鸡东万亩中药材种苗繁育及生产加工基地，积极引进大型医药集团，提高北药研发水平；加强梨树野生药材集散地建设，推动医药产业特色化、规模化、集群化发展。

促进企业裂变升级优存量。以打造“百亿航母、十亿板块、亿元群体”为目标，鼓励域内企业根据市场导向，调整产品结构，升级发展；兼并重组，扩张发展。重点推进珍宝岛药业集团金九药业大输液生产、乌苏里江药业刺五加中药饮片提取、新医圣蒲参盆腔泰、美城集团搬迁改造等40个裂变项目。进一步壮大规上企业群体，力争全年新增规上企业15户。支持普莱德、贝特瑞、浩市等企业上市融资。

发展矿业经济扩总量。把矿产资源开发及深加工作为调整工业结构的重要路径，加大石墨、硅藻土、大理岩等优势矿产资源的利用深度，努力把资源优势转化为产业优势。编制矿产资源及其深加工规划。加强资源勘查，促进新探矿权生成，激活盘活存量探矿权和采矿权。发挥市场机制作用，推动矿产资源整合，促进优势资源向优势企业、深加工企业配置。借助大专院校、科研院所力量，推进资源深度开发，延长产业链条，做大做强矿业经济。

(三)全力推进“两个转变”，在发展现代农业上实现新提升

实施品质提升工程。大力开展农业“三减”，绿色食品种植基地监测面积达到510万亩，推进种植业向“种得好、种得优”转变。建立鸡西农产品质量安全可追溯体系，打造100个“互联网＋农业”高标准示范基地。建设规模化高效节水灌溉示范片区，实施大型灌区及重点中型灌区的续建配套与节水改造工程，切实保障农业生产需求。

实施规模养殖工程。坚持“三进三退”、“四统一”，推进“两牛一猪一禽”规模化、标准化养殖。全年新建千头牛场3处、万头猪场2处、百万只鸡场1处、市级标准化示范场8处。推广青贮玉米、紫花苜蓿种植，饲草种植面积10万亩。

实施龙头带动工程。坚持种、养、加一体化发展，支持中粮米业、绿都集团、东粮集团等龙头企业做大做强做深，切实增强农畜产品加工转化能力。积极引进一批农畜产品深加工企业，壮大龙头企业群体。全年市级以上龙头企业发展到100家，带动产业化基地面积620万亩。

实施农民创业工程。进一步调整优化农业结构，扩大经济作物种植面积，大力发展棚室蔬菜、烤烟、树莓、食用菌、桑蚕、蜜蜂、河蟹等特色种植、养殖。推动一、二、三产融合发展，加强社会化服务体系建设，支持农民在种、养、加、流通、销售、服务等环节中就业创业，促进农民多领域增收。积极发展劳务经济，转移农村富余劳动力18万人。实施精准扶贫，5个村6000人到年底全部脱贫。

实施品牌营销工程。加大品牌整合力度，推动水稻、蜂产品、山产品等向虎林大米、兴凯湖大米等优势品牌集中。围绕绿色农产品、优质畜产品、特色山产品、名优水产品，争创一批著名商标、驰名商标、地理标志产品证明商标。建设50个淘宝村，推进农产品网上销售。推动我市绿色品牌产品进入省百家旗舰店、千家连锁店、万家主流超市，加快由“种得好”向“卖得好”转变。

(四)全力推进“旅贸牵动”，在激发三产活力上实现新提升

发展旅游业。以打造生态度假边境观光目的地为引领，构建“一核、一带、三区、五大基地”旅游布局，推动生态、红色、跨境三大旅游体系建设。围绕兴凯湖封闭管理，精心打造核心景区，做好虎头、珍宝岛5A级景区创建工作。积极引进战略投资者，开发精品旅游项目，推进虎头儿童水上乐园、东北(密山)革命根据地纪念园等项目建设。作好延点、延线、延时三篇文章，推动一季游向四季游转变。办好兴凯湖国际马拉松邀请赛、冬季冰雪汽车拉力赛等节庆活动，打造国内知名的旅游活动品牌。强化旅游线上线下宣传，推进“互联网＋旅游业”深度发展。加强与国内百强旅行社合作，搞好地接，提升接待水平，进一步提高鸡西旅游的知名度和影响力。

发展外经贸产业。以打造对俄国际通道和进出口加工基地为引领，充分利用国际国内两个市场、两种资源，推动对外经贸战略升级。积极推进跨境铁路建设，扩建、新建边民互市贸易区。坚持“出口抓加工、进口抓落地”，密山口岸突出粮食、液化石油气等产品进口，虎林口岸突出木材、牧草、中药材等产品进口，进一步扩大果蔬、山野菜、方便米饭等地工产品出口。积极引进战略投资者，建设规模化的地工产品出口加工项目和资源落地加工项目，加强对俄种植、森林采伐、建筑开发、境外加工等经济合作，培育壮大外经贸主体。加快虎林、鸡冠区电子商务园建设，促进跨境电子商务产业快速发展。

发展新兴服务业。科学编制物流业发展规划，推进泛华物流产业园区多式联运、冷链物流、仓储配送、电商快递、商务配套“五大中心”建设。积极发展银行、保险、证券等金融服务业。推进医养结合，加快发展健康养老服务业。提炼升华穆棱河文化、兴凯湖文化、北大荒文化，推进鸡西侵华日军罪证陈列馆、矿山历史文化馆、鸡西版画馆建设，积极发展文化创意产业。做实会展经济。推动房地产业健康发展。

(五)全力推进改革创新，在增强发展动力上实现新提升

深化改革增动力。加大行政审批制度改革力度，推进简政放权和行政审批项目公开，全面落实责任和权力两个清单，动态调整市级行政权力一次性告知单和流程图。加大公共资源市场化配置改革，推进兴凯湖景区封闭管理。深化投融资体制改革，支持企业上市融资，对接资本市场，扩大PPP模式应用领域。以农村土地承包经营权确权登记为重点，深入推进农村综合配套改革。

创新创业添活力。大力推进“大众创业、万众创新”，加快石墨产业园区孵化器、众创基地等创新平台建设，促进新技术、新产业、新业态蓬勃发展。全年新增科技型企业20户，新注册创业个体1万个。鼓励支持德元电器、浩市新能源等企业增强自主创新能力。发挥黑龙江工业学院、技师学院等院校作用，加快培养石墨、煤炭等产业基础人才。深入推进“兴凯湖智力行”活动，完善人才柔性引进机制。实施“互联网＋”三年行动计划，加强与中兴、阿里巴巴等互联网知名企业合作，建设电子政务云、互联网医疗预约会诊系统等示范项目，推动互联网与农业、旅游、医疗、政务等领域深度融合。

区域一体聚合力。做大县域经济。立足“产业强县、特色兴县”，加快密山、虎林、鸡东等园区建设，构建医药、绿色食品、旅游、外经贸等特色产业体系，增强县域经济综合实力。做强城区经济。按照全市产业布局，明确六区功能定位，坚持错位发展、协同发展，大上项目，培育壮大接续替代产业，增强发展内生动力，提升财政自给能力。加强市矿、场县、森地深度合作。支持龙煤集团解困发展，妥善分流安置富余人员。推动区矿、场县、森地融合发展，促进发展资源共享、产业项目共建、社会事业共办、城乡建设共兴，真正实现协同发展、共赢发展。

(六)全力推进城乡建设，在打造宜居环境上实现新提升

加强基础设施建设。做好新一轮城市总体规划修编，开展城市地下综合管廊建设和海绵城市建设规划编制。积极配合做好牡佳快铁鸡西段前期工作；完成密兴高速公路建设；拆建鸡虎高速团结站互通桥，确保9月份恢复通车。加快城市供水工程进度，6月份建成通水，确保百姓喝上放心水、优质水。推进永久性天然气场站建设，改造鸡冠老工业区供气管网，全部恢复原北钢、天元供气公司用户的供气；推进中燃并购赫阳燃气(原矿务局煤气厂)，分批启动原覆盖居民区供气。

统筹推动城乡建设。坚持城乡一体，协同发展，共同繁荣。改造提升中心城区，实施城市森林公园续建等工程，亮化建工街、腾飞桥等“五路二桥”，做好城市出入口、航空港和车站周边的绿化美化，完成祥光西路排水、中心区给水改造。努力逐步化解和解决城市历史遗留问题。打造特色小城镇，加快密山、虎林、鸡东中心镇和恒山、滴道等五城区建设，完善功能，提升品质，集聚人口；打造虎头、白鱼湾、平阳等一批旅游、商贸、文化特色名镇。建设美丽乡村，深入实施“三年行动计划”，创建美丽乡村示范村67个。

积极推进生态建设。完成兴凯湖生态环境保护重点项目，大力推进大气污染防治和主要污染物减排。积极开展生态修复，加强植树造林、退耕还林和水土流失治理，全年植树造林1.45万亩，治理水土流失4.5万亩。

提升城市管理水平。推进城市“四化”管理，坚持法治化，强化城市综合执法，深入开展“六治专项行动”，整治占道经营、乱贴乱画、乱倒垃圾、破坏公共设施等不文明行为。坚持专业化，加强城市管理人员的专业素养建设，提高专业化设备使用率，提升城市保洁和物业管理水平。坚持数字化，加快智慧城市建设，建成城市管理指挥、数据、决策中心智能管理平台，推进便民、便企应用系统上线运行。坚持人性化，推进文明执法、全民守法，全面提高执法水平和市民的文明素养，形成共建家园、呵护家园的良好氛围。

(七)全力推进民生改善，在增进人民福祉上实现新提升

保障和改善民生是政府一切工作的出发点和落脚点，是政府义不容辞的责任。我们将继续加大投入，着力提高社会保障水平，着力发展教育、文化、卫生、医疗、体育等社会事业，着力解决与人民群众生活息息相关的住房、供热、供水、供气、物价等热点难点问题，努力使我们的城市更温暖、更和谐，让广大人民群众生活得更幸福、更健康。

重点办好10件民生实事。(1)大力推进采煤沉陷区棚户区改造，新开工建设20379套，货币化安置1.1万户，让采沉区居民早日出棚进楼，实现安居梦。(2)加大贫困学生资助力度，全年资助3.1万人次；提高贫困大学生资助标准，最高一次性补助5000元，确保每名学生不因贫困而失学。(3)开工建设鸡冠区养老服务中心，切实提高养老保障水平。(4)建成市食品检验检测中心，提高食品安全技术支撑能力，为公众饮食安全提供有效保障。(5)开展团山水库、哈达水库周边环境治理，加强水源地水质保护，让居民喝上干净水、放心水。(6)推进农村分布式能源建设，累计改造1万户，提升农村居民生活品质。(7)开工建设多式联运货运枢纽工程，进一步完善城市功能。(8)实施兴国东路、冷家路、东环等16条街路改造维修工程，切实解决中心区基础设施落后问题。(9)穆棱河众兴大桥建成通车，让城市更畅通，出行更便捷。(10)开工建设鸡西市政务服务中心，提供更加便民便企的政务服务环境，打通服务群众“最后一公里”。

(八)全力推进安全稳定，在构建和谐鸡西上实现新提升

突出煤矿安全。从严落实安全生产责任，做到“五级五覆盖”、“五落实五到位”；从严地方煤矿复产验收标准，严厉追究违规责任；从严地方煤矿安全隐患排查整治，将隐患消灭在萌芽状态；从严开展打非治违专项活动，确保煤矿安全生产持续稳定。

强化地面安全。加大商场、学校等人员密集场所和危险化学品、烟花爆竹等行业安全隐患的整治力度，严厉查处酒驾、毒驾、“三超一疲劳”等交通违法行为。突出食品药品安全监管，严厉打击非法制售假冒伪劣食品行为。全力抓好特种设备、非煤矿山、森防消防、建筑施工等重点领域安全。

维护社会稳定。全面落实领导、部门信访包案责任制，坚持领导信访接待日和接访下访制度，切实解决群众反映突出问题。加强依法治访，严肃处理缠访、闹访行为。加强公安“四项建设”，打造立体化防控体系、网格化管理体系、法治化治理体系，强化网络虚拟社会管理，坚决遏制重大刑事案件发生。调动各方力量，切实维护边境安全稳定。

三、加强政府自身建设

面对转型发展的新任务，面对全市人民的新期待，我们将以饱满的热情、充足的干劲、务实的作风，高效理政，依法施政，从严治政，努力建设为民务实清廉政府。

坚持“四办”作风，建设实干政府。大力倡导“四办”作风，对事关发展的大项目、难事、要事，敢于担当，主动担当，勇于办；对工作中定下来的事，不折不扣，快速落实，马上办；对项目落地、企业需求、群众困难，不推不拖，限时解决，真正办；对市委、市政府的决策部署，见人、见事、见结果，加强检查，监督办。围绕服务转型发展，倾力打造专业素养、专业思维、专业方法的“三专”公务员队伍，提高执行力和担当能力。

坚持科学施政，建设效能政府。严格落实首问负责、一次性告知和限时办结制度，提高工作效率。发挥网上政务服务中心、市政务服务中心、企业投诉中心三大中心作用，进一步提高行政效能，为群众提供便捷优质的服务。严肃查处不作为、乱作为、慢作为和扰乱市场经济秩序行为，推进诚信鸡西建设，持续改善和优化经济发展环境。

坚持依法行政，建设法治政府。依法全面履行政府职能，法定职责必须为、法无授权不可为。完善公众参与、专家论证、风险评估、合法性审查、集体讨论的科学决策机制，积极推行政府法律顾问制度。自觉接受人大法律监督、政协民主监督和社会舆论监督。全面推进政务公开，让政府运行更加科学规范、阳光透明。

坚持从严治政，建设廉洁政府。严格遵守《廉洁自律准则》，自觉践行“三严三实”，坚持不懈改正“四风”。强化审计监督，加强政府投资的重大项目、公共资金、公共资源、国有资产的监管，依法依规推进政府采购、项目招投标、土地招拍挂等活动，严肃查处各类违法违纪行为。厉行勤俭节约，全年“三公”经费只减不增，最大限度降低行政成本。

各位代表，踏上新征程，实现新发展，是时代赋予我们的光荣使命和神圣职责。让我们在市委的坚强领导下，坚定信心，开拓进取，扎实工作，不断开创转型发展兴市富民新局面，努力创造鸡西人民幸福美好生活！
